# Clinical and epidemiological profile of Candidozyma auris notifications in a regional hospital: a descriptive study, Pernambuco, 2022–2023

**DOI:** 10.1590/S2237-962220v35e20251057.en

**Published:** 2026-05-08

**Authors:** Emanuela Batista Ferreira e Pereira, Marcielle dos Santos Santana, Felipe Almeida Sales, Raphael Alves da Silva, Camylla Carvalho de Melo, Katiuscia Araújo de Miranda Lopes, Reginaldo Gonçalves de Lima-Neto, Felicialle Pereira da Silva

**Affiliations:** 1 Universidade de Pernambuco Recife PE Brazil Universidade de Pernambuco, Recife, PE, Brazil; 2 Hospital Otávio de Freitas Programa de Residência em Enfermagem em Pneumologia Recife PE Brazil Hospital Otávio de Freitas, Programa de Residência em Enfermagem em Pneumologia, Recife, PE, Brazil; 3 Universidade Federal de Pernambuco Centro de Ciências Médicas Recife PE Brazil Universidade Federal de Pernambuco, Centro de Ciências Médicas, Recife, PE, Brazil

**Keywords:** Candydozyme Auris, Drug Resistance, Fungal, Clinical Epidemiology, Colonization, Hospital Infection Control Program, Candidozyma auris, Farmacorresistencia Fúngica, Epidemiología Clínica, Colonización, Programa de Control de Infecciones Hospitalarias

## Abstract

**Objective:**

To describe the clinical and epidemiological profile of *Candidozyma auris* colonization notifications in a regional trauma referral hospital of Pernambuco.

**Methods:**

Descriptive case series study using secondary data from the institutional notification system and electronic medical records. All individuals with confirmed *Candidozyma auris* colonization between January 2022 and July 2023 were included. Sociodemographic, clinical, care-related variables, and outcomes were analyzed using descriptive statistics.

**Results:**

A total of 44 individuals were included, predominantly male (77.3%) and <50 years old (56.8%). Most were admitted via transfer from other healthcare units (56.8%) and through the trauma emergency department (86.4%). Extensive use of invasive devices was observed (79.5%) along with high intra-hospital patient movement, with 65.9% of patients moving through four or more units. Concomitant bacterial infection occurred in 54.5% of cases, mainly caused by Staphylococcus spp., Pseudomonas spp., and Acinetobacter spp. The mean length of hospital stay was 55.7 days, and the mortality rate was 34.1%.

**Conclusion:**

*Candidozyma auris* colonization occurred in a context of high-complexity care, characterized by prolonged hospitalizations, intensive use of invasive devices, and multiple intra-hospital transfers. These findings underscore the need for targeted active surveillance, strict environmental control protocols, and integration among healthcare services to contain the spread of this emerging pathogen.

## Resumen

**Objetivo:**

Describir el perfil clínico y epidemiológico de las notificaciones de colonización por *Candidozyma auris* en un hospital regional de referencia en trauma de Pernambuco.

**Métodos:**

Estudio de serie de casos, de carácter descriptivo, con datos secundarios provenientes del sistema institucional de notificación y de historias clínicas electrónicas. Se incluyeron todas las personas con colonización confirmada por *Candidozyma auris* entre enero de 2022 y julio de 2023. Las variables sociodemográficas, clínicas y asistenciales, así como los desenlaces, se analizaron mediante estadística descriptiva.

**Resultados:**

Se incluyeron 44 personas, predominantemente del sexo masculino (77,3 %) y menores de 50 años (56,8 %). La mayoría fue admitida por transferencia de otras unidades de salud (56,8 %) y por emergencia de trauma (86,4 %). Se observó un uso extensivo de dispositivos invasivos (79,5 %) y una elevada rotación entre los sectores hospitalarios, con 65,9 % de los pacientes que transitaron por cuatro o más unidades. La infección bacteriana concomitante se observó en el 54,5 %, principalmente por Staphylococcus spp., Pseudomonas spp. y Acinetobacter spp. El tiempo medio de hospitalización fue de 55,7 días y la proporción de muertes fue de 34,1 %.

**Conclusión:**

La colonización por *Candidozyma auris* ocurrió en un contexto de alta complejidad asistencial, marcado por hospitalizaciones prolongadas, uso intensivo de dispositivos invasivos y múltiples transferencias intrahospitalarias. Estos hallazgos reforzaron la necesidad de vigilancia activa dirigida, de protocolos rigurosos de control ambiental e integración entre los servicios de salud para contener la diseminación de este patógeno emergente.

**Ethical aspects**

This research respected ethical principles, having obtained the following approval data:

Research Ethics Committee: Hospital da Restauração Governador Paulo Guerra

Opinion number: 6,044,810

Approval date: 8/5/2023

Certificate of Submission for Ethical Appraisal: 68881223.0.0000.5198

Informed Consent Form: Exempt.

## Introduction

The emergence of *Candidozyma auris *(C. auris) constitutes a contemporary challenge for epidemiological surveillance and the control of healthcare-associated infections, particularly in high-complexity hospital settings [1]. Since its identification, this fungus has been associated with outbreaks characterized by high transmission potential, prolonged environmental persistence, and a frequent profile of multidrug resistance to available antifungal agents. Unlike other Candida species, C. auris has a remarkable ability to colonize the skin and inanimate surfaces, which favors cross-transmission and the maintenance of transmission chains in healthcare settings [2].

In the international context, C. auris outbreaks have been described mainly in intensive care units (ICU) and in institutions that care for critically ill individuals, who are often subjected to multiple invasive procedures and prolonged hospital stays [3]. 

In Brazil, although records are still relatively recent, notifications have been increasing and are concentrated in large and high-complexity hospitals. Even so, national scientific production remains limited, particularly regarding the systematic description of healthcare contexts in which colonization occurs [4]. In this regard, descriptive studies based on case series play a strategic role by providing concrete evidence to support service organization, improve surveillance protocols, and guide institutional infection control policies.

Trauma referral hospitals represent particularly sensitive settings for the emergence of multidrug-resistant pathogens, as they concentrate critically ill individuals who are frequently subjected to emergency surgical procedures, intensive use of invasive devices, prolonged mechanical ventilation, and multiple intra-hospital transfers [5]. Understanding the clinical-epidemiological profile of individuals colonized by C. auris in this context is essential to guide targeted surveillance actions and more effective strategies to restrain its spread.

This study aimed to describe the clinical-epidemiological profile of notifications of C. auris colonization at a regional hospital in Pernambuco, to strengthen epidemiological surveillance actions, and to produce evidence applicable to healthcare services with a care profile similar to that of the Brazilian Unified Health System (*Sistema Único de Saúde*, SUS).

## Methods

### Study design

A descriptive case series study using secondary data from institutional epidemiological surveillance, conducted from January 2022 to July 2023.

### Setting

A large regional public hospital, a referral center for trauma and high-complexity emergency care in Pernambuco, with emergency services, a surgical center, intensive care units, and specialized wards, which predominantly serve users of the Brazilian Unified Health System (SUS).

### Participants

All individuals with confirmed colonization by *Candidozyma auris* between January 2022 and July 2023 were included in the study. Suspected cases or those with inconclusive laboratory results were not considered.

### Data source

Cases were identified through the institutional notification system for microorganisms of epidemiological relevance, under the responsibility of the Healthcare-Associated Infection Control Committee (*Comissão de Controle de Infecção Relacionada à Assistência à Saúde*, CCIH), complemented by the review of electronic medical records.

### Laboratory confirmation

Colonization by C. auris was confirmed by microbiological culture, identification by matrix-assisted laser desorption ionization time-of-flight mass spectrometry (MALDI-TOF), and/or the automated VITEK 2 system, according to institutional availability during the study period.

### Data collection

Sociodemographic variables, healthcare-related characteristics, use of invasive devices, presence of comorbidities, occurrence of concomitant bacterial infection, isolated microorganisms, and clinical outcomes were collected using a standardized form.

### Data analysis

Data were analyzed using descriptive statistics, with presentation of absolute and relative frequencies for categorical variables, and measures of central tendency and dispersion for numerical variables.

## Results

The sample included 44 patients colonized with *Candida auris* between January 2022 and July 2023. The main type of hospital admission observed was transfer from other healthcare facilities (56.8%), followed by admissions via the Mobile Emergency Care Service/Fire Department (27.3%) and by spontaneous demand (15.9%), with the trauma emergency department as the main entry point (86.4%) (Table 1). The mean age of the patients was 48.2 years, with a predominance of males (77.3%). A total of 68.2% of patients had no previous comorbidities, while 31.8% had chronic conditions, mainly diabetes and hypertension.

**Table 1:** Absolute and relative frequencies of the sociodemographic characteristics of individuals colonized by *Candidozyma auris* in a regional hospital. Pernambuco, 2022–2023 (n=44)

Variable	n (%) 44 (100.0)
**Age group **(years)	
<50	25 (56.8)
>50	19 (43.2)
Sex	
Male	34 (77.3)
Female	10 (22.7)
**Place of Residence**	
Metropolitan region of Recife	24 (54.5)
Outside the metropolitan region of Recife	20 (45.5)
Origin	
Spontaneous demand	7 (15.9)
Transfer	25 (56.8)
Mobile Emergency Care Service/Fire Department	12 (27.3)
**Type of service entry**	
Clinical emergency	6 (13.6)
Trauma emergency	38 (86.4)

Invasive devices were used in 79.5% of patients, particularly central venous catheters, indwelling urinary catheters, and mechanical ventilation. A total of 59.1% used two or more devices during hospitalization. Surgical procedures were performed in 70.5% of cases, generally related to trauma care. Concomitant bacterial infection occurred in 54.5% of patients, with higher frequencies of Staphylococcus spp. (27.3%), Pseudomonas spp. (20.5%), Acinetobacter spp. (18.2%), and Candida spp. (11.4%).

At the time of testing positive for C. auris, 43.2% of patients were in the ICU and 38.6% were in hospital wards. Analysis of intra-hospital trajectories showed high patient movement, with 65.9% of patients moving through four or more sectors (Table 2). The mean length of hospital stay was 55.7 days, and the mean ICU stay was 35.1 days. Regarding clinical outcomes, 61.4% were discharged from the hospital, 34.1% died, and 4.5% were transferred to other facilities (Table 2).

**Table 2:** Absolute and relative frequencies of the clinical characteristics of individuals colonized by *Candidozyma auris* in a regional hospital. Pernambuco, 2022–2023 (n=44)

Variable	n (%) 44 (100.0)
Comorbidity	
Yes	14 (31.8)
No	30 (68.2)
**Number of invasive devices used during the hospital stay**	
One	8 (18.2)
More than one device	35 (79.5)
None	1 (2.3)
**Other cultures performed during hospitalization**	
Blood culture	38 (86.4)
Urine culture	6 (13.6)
**Associated bacterial infection**	
Yes	24 (54.5)
No	20 (45.5)
**Type of isolated microorganisma**	
Staphylococcus	12 (27.3)
Pseudomonas	9 (20.5)
Acinetobacter	8 (18.2)
Candida	5 (11.4)
Proteus	4 (9.1)
Escherichia	2 (4.5)
Providencia	2 (4.5)
Burkholderia	1 (2.3)
Klebsiella	1 (2.3)
Morganella	1 (2.3)
**Hospital unit at the time of testing positive**	
Intensive Care Unit	19 (43.2)
Wards	17 (38.6)
Transition units (Post-anesthesia care unit, Clinical emergency, and Trauma emergency units)	8 (18.1)
**Number of hospital units transferred through**	
1–3	15 (34.1)
>4	29 (65.9)
**Length of hospital stay **(days)	
8–30	13 (29.5)
31–60	16 (36.4)
>61	15 (34.1)
**Length of stay in the Intensive Care Unit **(days)	
None	9 (20.5)
2–30	13 (29.5)
31–60	15 (34.1)
>61	7 (15.9)
Outcome	
Hospital discharge	27 (61.4)
Transfer	2 (4.5)
Death	15 (34.1)

^a^Due to multiple isolations, the sum of frequencies exceeds the total.

## Discussion

The findings of this study showed that C. auris colonization occurred in a healthcare setting characterized by high complexity, combining prolonged hospitalizations, intensive use of invasive devices, and high intra-hospital patient movement. The convergence of these factors at a regional trauma referral hospital underscores the need for institutions with a similar profile to adopt specific, systematic active surveillance strategies, rather than relying solely on passive notifications [6]. 

More than half of the patients presented concomitant bacterial infections during hospitalization, with Staphylococcus spp., Pseudomonas spp., and Acinetobacter spp. being most prominent, frequently associated with high antimicrobial pressure scenarios [7]. The coexistence of C. auris colonization with bacterial infections highlighted that these cases occur within a hospital ecosystem characterized by intense antimicrobial exposure and a high risk of selecting multidrug-resistant microorganisms [8].

The observed mortality rate was approximately one-third of cases. Although it is not possible to establish a direct causal relationship between C. auris colonization and death, given the high clinical severity of the studied population, this finding reinforces the need for focused attention to this group and the strengthening of infection control and surveillance measures [9]. Most admissions occurred via transfer from other healthcare facilities and through the trauma emergency department, which emphasizes the clinical complexity of the population served and the potential introduction of the microorganism through interinstitutional circulation of colonized individuals [10]. Hospital stays were prolonged, with a mean length exceeding 50 days, reflecting the clinical severity of the cases and the complexity of the required care. The prolonged hospitalization constituted an additional risk factor for the acquisition and maintenance of *C. auris *[11]. The high frequency of invasive device use observed deserves special attention, as these devices represent important entry points and reservoirs for microorganisms in the human body and serve as surfaces conducive to biofilm formation [12]. The presence of catheters, mechanical ventilation, and urinary catheters increases exposure to potentially contaminated environments and heightens vulnerability to colonization [8]. These findings are consistent with the international literature, which has described a strong association between C. auris and healthcare settings characterized by life support, invasive procedures, and frequent use of broad-spectrum antimicrobials [12–13].

The high intra-hospital patient movement increases the potential for microorganism dissemination across different environments and patient populations, posing additional challenges for epidemiological surveillance [14]. 

The occurrence of concomitant bacterial infections indicated that C. auris colonization occurs within a hospital ecosystem whose surveillance should not be considered in isolation. Still, it integrated into broader strategies for monitoring microbial resistance and promoting rational antimicrobial use [15].

From an operational perspective, the results reinforced the importance of active case-finding as a central tool for the early detection of C. auris colonization. Timely identification allows for the immediate implementation of contact precautions, appropriate isolation, enhanced environmental cleaning, and intensified educational interventions for healthcare teams [16]. 

The high proportion of admissions via transfer from other facilities highlighted the importance of interinstitutional communication in preventing the spread of C. auris [14,15]. 

As study limitations, the descriptive design and the use of secondary data—subject to incomplete records—were noted, along with the small sample size and the absence of molecular characterization of isolates, which could have provided deeper insight into transmission chains. Despite these limitations, this study expanded knowledge on the clinical-epidemiological profile of C. auris colonization in a Brazilian public hospital, reinforcing the importance of active surveillance and screening in critically ill patients. The findings pointed to the need for standardization of identification and notification criteria within the SUS, as well as the strengthening of infection prevention and control measures, integrating clinical, epidemiological, and laboratory data.

## Data Availability

Individual-level data containing potentially identifiable information is not publicly available for ethical reasons.
