# Supplementary material for: Clinical and epidemiological profile of Candidozyma auris notifications in a regional hospital: a descriptive study, Pernambuco, 2022–2023
Source: Epidemiol Serv Saude. 2026 May 8;35:e20251057. doi: 10.1590/S2237-962220v35e20251057.en (PMC13686417; doi:10.1590/S2237-962220v35e20251057.en)
Supplement: Translation

# Translation: Perfil clínico-epidemiológico das notificações de Candidozyma auris em hospital regional: estudo descritivo, Pernambuco, 2022-2023

**DOI:** 10.1590/S2237-962220v35e20251057.pt

**Authors:** Emanuela Batista Ferreira e Pereira, Marcielle dos Santos Santana, Felipe Almeida Sales, Raphael Alves da Silva, Camylla Carvalho de Melo, Katiuscia Araújo de Miranda Lopes, Reginaldo Gonçalves de Lima-Neto, Felicialle Pereira da Silva

**Affiliations:** 1 Universidade de Pernambuco Recife PE Brasil Universidade de Pernambuco, Recife, PE, Brasil; 2 Hospital Otávio de Freitas Programa de Residência em Enfermagem em Pneumologia Recife PE Brasil Hospital Otávio de Freitas, Programa de Residência em Enfermagem em Pneumologia, Recife, PE, Brasil; 3 Universidade Federal de Pernambuco Centro de Ciências Médicas Recife PE Brasil Universidade Federal de Pernambuco, Centro de Ciências Médicas, Recife, PE, Brasil

**Keywords:** Candydozima Auris, Farmacorresistência fúngica, Epidemiologia Clínica, Colonização, Programa de Controle de Infecção Hospitalar

## Abstract

**Objetivo:**

Descrever o perfil clínico-epidemiológico das notificações de colonização por Candidozyma auris em um hospital regional de referência em trauma em Pernambuco.

**Métodos:**

Estudo de série de casos, de caráter descritivo, com dados secundários provenientes do sistema institucional de notificação e de prontuários eletrônicos. Incluíram-se todas as pessoas com colonização confirmada por Candidozyma auris entre janeiro de 2022 e julho de 2023. As variáveis sociodemográficas, clínicas, assistenciais e os desfechos foram analisados por estatística descritiva.

**Resultados:**

Foram incluídas 44 pessoas, predominantemente do sexo masculino (77,3%) e <50 anos (56,8%). A maioria foi admitida por transferência de outras unidades de saúde (56,8%) e pela emergência de trauma (86,4%). Observou-se uso extensivo de dispositivos invasivos (79,5%) e elevada rotatividade entre setores hospitalares, com 65,9% de pacientes que transitaram por quatro ou mais unidades. Infecção bacteriana concomitante ocorreu em 54,5%, principalmente por Staphylococcus spp., Pseudomonas spp. e Acinetobacter spp. O tempo médio de hospitalização foi de 55,7 dias, e a proporção de óbitos foi de 34,1%.

**Conclusão:**

A colonização por Candidozyma auris ocorreu em contexto de alta complexidade assistencial, marcado por internações prolongadas, uso intensivo de dispositivos invasivos e múltiplas transferências intra-hospitalares. Esses achados reforçaram a necessidade de vigilância ativa dirigida, protocolos rigorosos de controle ambiental e integração entre serviços de saúde para contenção da disseminação desse patógeno emergente.

**Aspectos éticos**

A presente pesquisa respeitou os princípios éticos, obtendo os seguintes dados de aprovação:

Comitê de ética em pesquisa: Hospital da Restauração Governador Paulo Guerra

Número do parecer: 6.044.810

Data de aprovação: 8/5/2023

Certificado de apresentação de apreciação ética: 68881223.0.0000.5198

Registro do consentimento livre e esclarecido: Dispensado.

## Introdução

A emergência de *Candidozyma auris *(C. auris) constitui um dos desafios contemporâneos para a vigilância epidemiológica e o controle de infecções relacionadas à assistência à saúde, sobretudo em ambientes hospitalares de alta complexidade [1]. Desde sua identificação, o fungo tem sido associado a surtos caracterizados por elevada capacidade de disseminação, persistência ambiental prolongada e perfil frequente de multirresistência aos antifúngicos disponíveis. Diferentemente de outras espécies do gênero Candida, C. auris apresenta notável capacidade para colonizar a pele e superfícies inanimadas, o que favorece a transmissão cruzada e a manutenção de cadeias de disseminação nos serviços de saúde [2].

No cenário internacional, surtos de C. auris têm sido descritos, principalmente em unidades de terapia intensiva (UTI) e em instituições que concentram pessoas em estado crítico, submetidas a múltiplos procedimentos invasivos e a longos períodos de internação [3]. 

No Brasil, embora os registros ainda sejam relativamente recentes, as notificações vêm aumentando e concentram-se em hospitais de grande porte e de alta complexidade. Mesmo assim, a produção científica nacional ainda é restrita, especialmente no que se refere à descrição sistematizada dos contextos assistenciais nos quais ocorre a colonização [4]. Nesse sentido, estudos descritivos baseados em séries de casos desempenham papel estratégico ao fornecer subsídios concretos para a organização dos serviços, o aprimoramento dos protocolos de vigilância e a orientação das políticas institucionais de controle de infecção.

Hospitais de referência em trauma representam cenários particularmente sensíveis para a emergência de patógenos multirresistentes, pois concentram pessoas em estado crítico, frequentemente submetidas a procedimentos cirúrgicos de urgência, uso intensivo de dispositivos invasivos, ventilação mecânica prolongada e múltiplas transferências intra-hospitalares [5]. Compreender o perfil clínico-epidemiológico de pessoas colonizadas por C. auris, nesse contexto, é fundamental para orientar ações de vigilância direcionadas e estratégias mais eficazes de contenção da disseminação.

Este estudo teve como objetivo descrever o perfil clínico-epidemiológico das notificações de colonização por C. auris em um hospital regional, localizado em Pernambuco, para o fortalecimento das ações de vigilância epidemiológica e a produção de evidências aplicáveis a serviços de saúde com perfil assistencial semelhante ao Sistema Único de Saúde (SUS).

## Métodos

### Delineamento

Estudo de série de casos, de caráter descritivo, com utilização de dados secundários de vigilância epidemiológica institucional, no período de janeiro de 2022 a julho de 2023.

### Contexto

Hospital público regional de grande porte, referência para o atendimento de trauma e de urgências de alta complexidade em Pernambuco, com serviços de emergência, centro cirúrgico, unidades de terapia intensiva e enfermarias especializadas, os quais atendem predominantemente usuários do SUS.

### Participantes

Foram incluídas no estudo todas as pessoas com colonização confirmada por *Candidozyma auris* no período de janeiro de 2022 a julho de 2023. Casos suspeitos ou com resultados laboratoriais inconclusivos não foram considerados.

### Fonte de dados

Os casos foram identificados por meio do sistema institucional de notificação de microrganismos de relevância epidemiológica, sob responsabilidade da Comissão de Controle de Infecção Relacionada à Assistência à Saúde (CCIH), complementados pela revisão de prontuários eletrônicos.

### Confirmação laboratorial

A colonização por C. auris foi confirmada por cultura microbiológica, identificação por espectrometria de massas (MALDI-TOF) e/ou sistema automatizado VITEK 2, conforme disponibilidade institucional no período.

### Coleta de dados

Foram coletadas variáveis sociodemográficas, características assistenciais, uso de dispositivos invasivos, presença de comorbidades, ocorrência de infecção bacteriana concomitante, microrganismos isolados e desfecho clínico, com utilização de formulário padronizado.

### Análise dos dados

Os dados foram analisados por estatística descritiva, com apresentação de frequências absolutas e relativas para variáveis categóricas, e de medidas de tendência central e de dispersão para variáveis numéricas.

## Resultados

A amostra incluiu 44 pacientes com colonização por *Candida auris* entre janeiro de 2022 e julho de 2023. A principal forma de admissão hospitalar observada foi a transferência de outras unidades de saúde (56,8%), seguida por ingressos via Serviço de Atendimento Móvel de Urgência/Corpo de Bombeiros (27,3%) e por demanda espontânea (15,9%), com o setor de emergência de trauma como principal porta de entrada (86,4%) (Tabela 1). A idade média dos pacientes foi de 48,2 anos, com predomínio do sexo masculino (77,3%). 68,2% dos pacientes não apresentava comorbidades prévias, enquanto 31,8% possuíam condições crônicas, principalmente diabetes e hipertensão.

**Tabela 1:** Frequências absolutas e relativas das características sociodemográficas de pessoas colonizadas por *Candydozima auris* em hospital regional. Pernambuco, 2022-2023 (n=44)

Variável	n (%) 44 (100,0)
**Faixa etária **(anos)	
<50	25 (56,8)
>50	19 (43,2)
Sexo	
Masculino	34 (77,3)
Feminino	10 (22,7)
**Local de residência**	
Região metropolitana do Recife	24 (54,5)
Fora da região metropolitana do Recife	20 (45,5)
Procedência	
Demanda espontânea	7 (15,9)
Transferência	25 (56,8)
Serviço de Atendimento Móvel de Urgência/Corpo de Bombeiros	12 (27,3)
**Tipo de entrada no serviço**	
Emergência clínica	6 (13,6)
Emergência de trauma	38 (86,4)

O uso de dispositivos invasivos foi observado em 79,5% dos pacientes, com destaque para cateter venoso central, sonda vesical de demora e ventilação mecânica. 59,1% utilizaram dois ou mais dispositivos durante a internação. Procedimentos cirúrgicos foram realizados em 70,5% dos casos, em geral relacionados ao atendimento do trauma. Infecção bacteriana concomitante ocorreu em 54,5% dos pacientes, com maior frequência de Staphylococcus spp. (27,3%), Pseudomonas spp. (20,5%), Acinetobacter spp. (18,2%) e Candida spp. (11,4%).

No momento da positividade para C. auris, 43,2% dos pacientes encontravam-se em UTI, e 38,6% em enfermarias. A análise dos trajetos intra-hospitalares evidenciou elevada rotatividade, com 65,9% de pacientes que transitaram por quatro ou mais setores (Tabela 2). O tempo médio de hospitalização foi de 55,7 dias, e a permanência média em UTI foi de 35,1 dias. Quanto aos desfechos clínicos, 61,4% receberam alta hospitalar, 34,1% evoluíram para óbito e 4,5% foram transferidos para outras unidades (Tabela 2).

**Tabela 2:** Frequências absolutas e relativas das características clínicas de pessoas colonizadas por *Candydozima auris *em hospital regional. Pernambuco, 2022-2023 (n=44)

Variável	n (%) 44 (100,0)
Comorbidades	
Sim	14 (31,8)
Não	30 (68,2)
**Número de dispositivos invasivos instalados durante tempo de permanência na unidade hospitalar**	
Um	8 (18,2)
Mais de um dispositivo	35 (79,5)
Não usou	1 (2,3)
**Outras culturas realizadas durante o internamento**	
Hemocultura	38 (86,4)
Urocultura	6 (13,6)
**Infecção bacteriana associada**	
Sim	24 (54,5)
Não	20 (45,5)
**Tipo de microorganismo isoladoa**	
Staphylococcus	12 (27,3)
Pseudomonas	9 (20,5)
Acinetobacter	8 (18,2)
Candida	5 (11,4)
Proteus	4 (9,1)
Escherichia	2 (4,5)
Providência	2 (4,5)
Burkholderia	1 (2,3)
Klebsiella	1 (2,3)
Morganella	1 (2,3)
**Setor de internamento no momento da positividade**	
Unidade de Terapia Intensiva	19 (43,2)
Enfermarias	17 (38,6)
Setores de transição (Serviço de recuperação pós-anestésica, Emergência clínica e Emergência de trauma)	8 (18,1)
**Número de setores hospitalares percorridos**	
1-3	15 (34,1)
>4	29 (65,9)
**Tempo de internação hospitalar **(dias)	
8-30	13 (29,5)
31-60	16 (36,4)
>61	15 (34,1)
**Tempo de internação em Unidade de Terapia Intensiva **(dias)	
Nenhum	9 (20,5)
2-30	13 (29,5)
31-60	15 (34,1)
>61	7 (15,9)
Desfecho	
Alta hospitalar	27 (61,4)
Transferência	2 (4,5)
Óbito	15 (34,1)

^a^Considerando-se a ocorrência de isolamentos múltiplos, a soma das frequências é superior ao total.

## Discussão

Os achados desta pesquisa evidenciaram que a colonização por C. auris ocorreu em um cenário assistencial caracterizado por alta complexidade, no qual se conjugam internações prolongadas, uso intensivo de dispositivos invasivos e elevada rotatividade intra-hospitalar. A convergência desses fatores no contexto de um hospital regional de referência em trauma reforça a necessidade de que instituições com perfil semelhante adotem estratégias específicas e sistematizadas de vigilância ativa, em vez de se basearem exclusivamente em notificações passivas [6]. 

Mais da metade das pessoas apresentou infecção bacteriana concomitante durante o período de internação, com destaque para microrganismos como Staphylococcus spp., Pseudomonas spp. e Acinetobacter spp., frequentemente associados a cenários de elevada pressão antimicrobiana [7]. A coexistência de colonização por C. auris com infecções bacterianas reforçou a inserção desses casos em um ecossistema hospitalar caracterizado por intensa exposição a antimicrobianos e elevado risco de seleção de microrganismos multirresistentes [8].

A proporção de óbitos observada aproximou-se de um terço dos casos. Embora não seja possível estabelecer relação causal direta entre a colonização por C. auris e o óbito, em razão da elevada gravidade clínica da população estudada, esse achado reforçou a necessidade de atenção a esse grupo e de fortalecimento das ações de vigilância e de controle de infecção [9]. A maioria das admissões ocorreu por transferência de outras unidades de saúde e pela porta de entrada da emergência de trauma, o que reforçou a complexidade clínica da população atendida e a possibilidade de introdução do microrganismo por meio da circulação interinstitucional de pessoas colonizadas [10]. A permanência hospitalar foi prolongada, com média superior a 50 dias, o que refletiu a gravidade clínica dos casos e a complexidade do cuidado demandado. A longa duração das internações constituiu fator adicional de risco para a aquisição e a manutenção da colonização por *C. auris *[11]. A elevada frequência de utilização de dispositivos invasivos observada merece destaque, já que eles constituem importantes vias de entrada e de manutenção de microrganismos no corpo humano, além de funcionarem como superfícies propícias à formação de biofilmes [12]. A presença de cateteres, de ventilação mecânica e de sondas vesicais amplia a exposição a ambientes potencialmente contaminados e aumenta a vulnerabilidade à colonização [8]. Esses achados estão em consonância com a literatura internacional, que descreveu forte associação entre C. auris e contextos assistenciais marcados por suporte intensivo à vida, por procedimentos invasivos e por uso frequente de antimicrobianos de amplo espectro [12-13].

A expressiva rotatividade intra-hospitalar amplia a possibilidade de disseminação do microrganismo entre diferentes ambientes e populações assistidas, o que impõe desafios adicionais à vigilância epidemiológica [14]. 

A ocorrência de infecções bacterianas concomitantes indicou que a colonização por C. auris se inseriu em um ecossistema hospitalar cuja vigilância não deve ser compreendida de forma isolada, mas integrada a estratégias mais amplas de monitoramento da resistência microbiana e de uso racional de antimicrobianos [15].

Do ponto de vista operacional, os resultados reforçaram a importância da busca ativa como ferramenta central para a detecção precoce de casos de colonização por C. auris. A identificação oportuna possibilita a adoção imediata de precauções de contato, o isolamento adequado, o reforço da higienização ambiental e a intensificação de ações educativas junto às equipes assistenciais [16]. 

A elevada proporção de admissões por transferência de outras unidades evidenciou a importância da comunicação interinstitucional na prevenção da disseminação de *C. auris *[14,15]. 

Como limitações do estudo, destacaram-se o delineamento descritivo e o uso de dados secundários, sujeitos à incompletude de registros, além do tamanho amostral restrito e da ausência de caracterização molecular dos isolados, a qual poderia aprofundar a compreensão das cadeias de transmissão. Apesar das limitações do delineamento, este estudo ampliou o conhecimento sobre o perfil clínico-epidemiológico da colonização por C. auris em um hospital público brasileiro, o que reforçou a importância da vigilância ativa e do rastreamento em pacientes críticos. Os achados apontaram para a necessidade de padronização de critérios de identificação e de notificação no âmbito do SUS, além de fortalecimento das ações de prevenção e de controle de infecções, com integração entre dados clínicos, epidemiológicos e laboratoriais. 

## Data Availability

Os dados individuais com informações potencialmente identificáveis não estão depositados publicamente por motivos éticos.
